# Global transcriptomic analysis suggests carbon dioxide as an environmental stressor in spaceflight: A systems biology GeneLab case study

**DOI:** 10.1038/s41598-018-22613-1

**Published:** 2018-03-08

**Authors:** Afshin Beheshti, Egle Cekanaviciute, David J. Smith, Sylvain V. Costes

**Affiliations:** 1 Wyle Labs, Space Biosciences Division, NASA Ames Research Center, Mountain View, CA USA; 2 Universities Space Research Association, Space Biosciences Division, NASA Ames Research Center, Mountain View, CA USA; 3 NASA, Space Biosciences Division, NASA Ames Research Center, Mountain View, CA USA

## Abstract

Spaceflight introduces a combination of environmental stressors, including microgravity, ionizing radiation, changes in diet and altered atmospheric gas composition. In order to understand the impact of each environmental component on astronauts it is important to investigate potential influences in isolation. Rodent spaceflight experiments involve both standard vivarium cages and animal enclosure modules (AEMs), which are cages used to house rodents in spaceflight. Ground control AEMs are engineered to match the spaceflight environment. There are limited studies examining the biological response invariably due to the configuration of AEM and vivarium housing. To investigate the innate global transcriptomic patterns of rodents housed in spaceflight-matched AEM compared to standard vivarium cages we utilized publicly available data from the NASA GeneLab repository. Using a systems biology approach, we observed that AEM housing was associated with significant transcriptomic differences, including reduced metabolism, altered immune responses, and activation of possible tumorigenic pathways. Although we did not perform any functional studies, our findings revealed a mild hypoxic phenotype in AEM, possibly due to atmospheric carbon dioxide that was increased to match conditions in spaceflight. Our investigation illustrates the process of generating new hypotheses and informing future experimental research by repurposing multiple space-flown datasets.

## Introduction

Comprehensive analysis of molecular signatures, such as transcriptional profiling, has become a standard technique in space biosciences and typically generates more extensive data than is required for the specific topic of investigation. Making all spaceflight data publicly accessible ensures that biological experiments can be repurposed to answer novel research questions and generate hypotheses. Therefore, the GeneLab open science platform (genelab.nasa.gov) was created to store raw molecular “omics” data from ground and spaceflight biology experiments supported by NASA. Here we present a case study using GeneLab datasets generated from ground controls associated with multiple rodent spaceflight datasets. Our overarching aim is to generate a hypothesis to drive future spaceflight rodent research and examine the potential impact of one known confounding factor in spaceflight, i.e. the environment in the animal habitat.

We proceeded by incorporating multiple, independent publicly available transcriptomic datasets from spaceflight experiments. Investigating spaceflight-induced changes in the transcriptome involves sending model organisms to orbit, such as rodents on the space shuttle (Space Transportation System program, STS), on satellites such as Bion-M1 (BF), or on the International Space Station (ISS). In these experiments multiple aspects of the environment are collectively altered. NASA space flown rodents are housed in a specific type of cage, called the Animal Enclosure Module (AEM). Within an AEM, animals will experience gravitational changes ranging from hypergravity during launch and landing to microgravity in orbit while simultaneously exposed to higher levels of ionizing radiation than found on Earth^1^.

**Figure 1:**
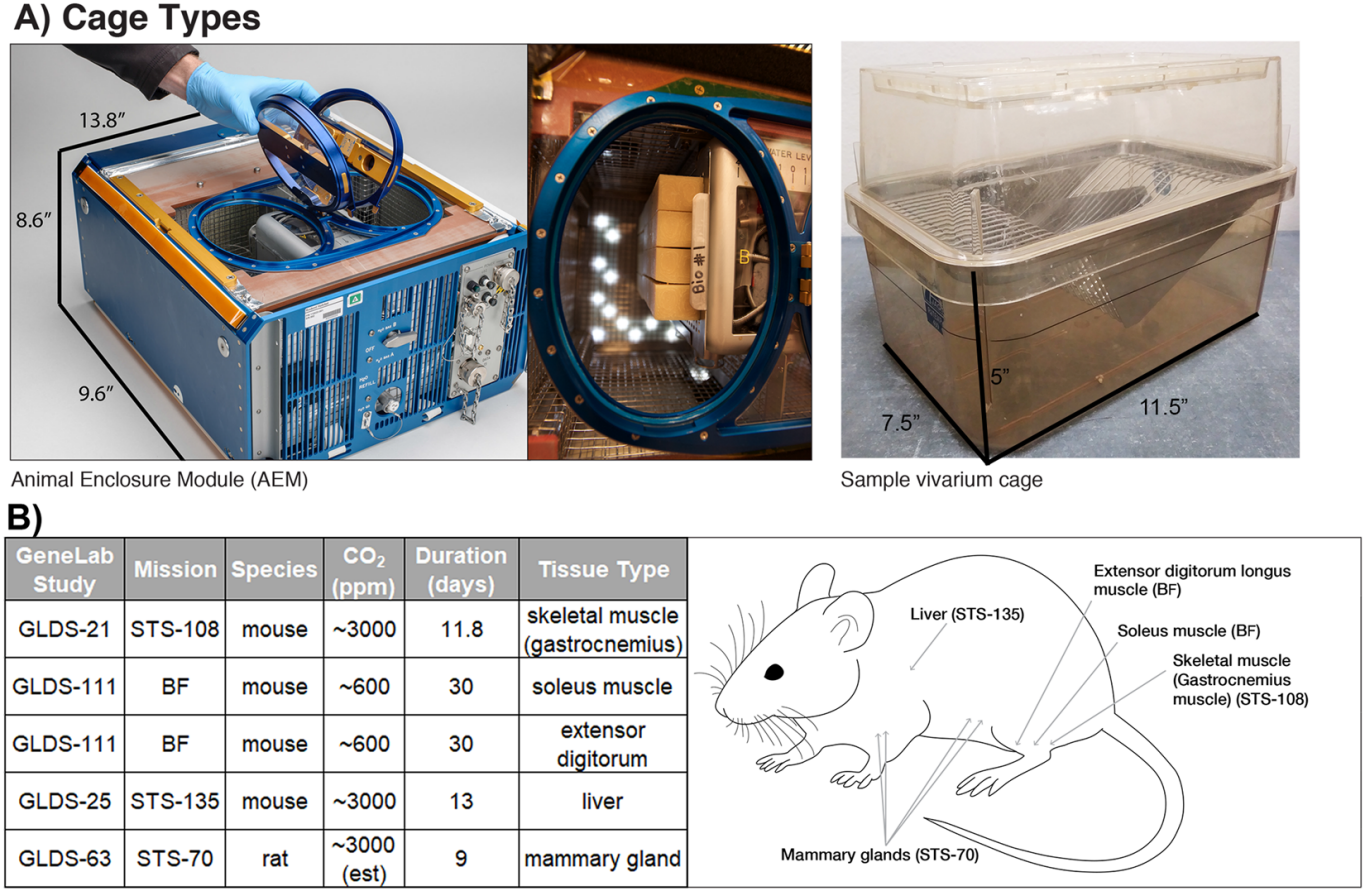
Illustration of the AEM and vivarium cages and GeneLab datasets used for analysis. (**A**) Images and dimensions of the both the AEM and vivarium cages used to house rodents. The upper two photos of the AEM cage was provided by NASA (Credits: NASA/Dominic Hart and https://www.nasa.gov/ames/research/space-biosciences/rodent-research-1). The vivarium cage photo was taken in our laboratory. (**B**) The GeneLab datasets which were used for analysis including the information on rodent species, the specific tissue type and its approximate location in a rodent, duration of experiment, and CO_2_ levels in the AEM cage.

Selecting appropriate controls for such multifactorial experiments is therefore complicated. The most frequently used experimental design is to keep all environmental conditions the same except for the flight by containing rodents in an AEM on spacecraft and using the same type of AEM hardware for ground controls^2^. An alternative and complementary approach is using regular vivarium cages for housing rodents as controls. AEM has been used as the standard rodent enclosure without major modifications in spaceflight experiments from STS^2^ to Bion-M1 (BF)^3^, and its more modern version called the Habitat module of the Rodent Research Hardware System is currently used on the ISS. Both AEM and vivarium housing follow the standard guidelines for laboratory animal care, which require at least 15 square inches per >25 g adult rodent (NASA Johnson Space Center Animal Care and Use Handbook). AEM can either contain up to 10 mice in two compartments (5 mice per compartment) or 6 rats maximum (3 rats per compartment). This is in comparison to the vivarium cages which can house either 5 mice, or 2 rats maximum in a single compartment (depending on the rat’s mass). The AEM has a combined larger surface area per rodent, because it includes climbable walls throughout the enclosure (Fig. 1A). Rodents housed in AEM and vivarium cages are typically kept on the same light/dark cycle and the air has the same oxygen concentration. However, CO_2_ concentration in AEM ground controls replicates CO_2_ concentration on spacecraft^2,4^, which tends to be up to an order of magnitude higher than on Earth. In general, CO_2_ concentration on ground and therefore, in vivarium cages is approximately 300 ppm, while on the space shuttle and in matched ground AEM it reaches up to 3000 ppm^5^. Notably, it was lower in the AEM controls for the Bion-M1 satellite study: 682 ppm on average with 718 ppm standard deviation (range: 201–2096 ppm)^4^. Interestingly, previous studies on different CO_2_ conditions on human health have revealed major impact on cognitive functions. Specifically, it was observed that a 400 ppm increase in CO_2_ levels results in a 21% drop in cognitive scores^6^ and specific research related to the increased CO_2_ levels on the ISS have shown increased incidence of headaches with astronauts on the ISS^7^.

Here we analyze the impact of changing only the habitat, from the classic vivarium cage to the spaceflight-matched AEM, using ground control datasets located in the GeneLab depository (Fig. 1B). We combine multiple experiments and species (C57BL/6 mice (*Mus musculus*) and rats (*Rattus norvegicus*)) to increase the certainty that any common changes found across these datasets are consistent and reproducible. Additionally, by utilizing multiple tissues (muscle, breast and liver) in our analysis we are able to define a global systematic biological response to the different housing conditions. Finally, by using a previously-established systems biology approach to combine multiple analysis methods^8^ we are able to uncover a statistical consensus on molecular pathways being specifically modified by the change in animal housing.

## Materials and Methods

### Data from GeneLab Platform

Processing of animal tissues, RNA extraction, and microarray details were previously reported by NASA funded investigators associated with each dataset^2,3,9,10^. Our results are based on openly-available data housed in the NASA GeneLab platform (genelab.nasa.gov). Although some of these datasets can also be found on NCBI’s Gene Expression Omnibus (GEO), not all NASA funded transcriptomic data will be available on GEO. In general, the GeneLab platform will provide the most publicly available comprehensive NASA related “-omics” data. We analyzed the following datasets from GeneLab: GLDS-21, GLDS-111, GLDS-25, and GLDS-63 (Fig. 1B). These four datasets were chosen because they are the only datasets available for rodents on GeneLab that have both the AEM and vivarium controls. For each dataset we only performed analysis between the AEM and vivarium controls. The following replicates were available for each dataset for both conditions: for GLDS-21 there were 4 AEM controls and 5 vivarium controls, for GLDS-111 there were 3 AEM controls and 3 vivarium controls for both Soleus and Extensor digitorum muscles, for GLDS-25 there were 5 AEM controls and 6 vivarium controls, and for GLDS-63 there were 3 AEM controls and 4 vivarium controls. GLDS-25 and -21 datasets were obtained from 9 to 11-week old, female C57BL/6 mice. GLDS-63 dataset was derived from female pregnant Sprague-Dawley rats. GLDS-111 dataset was procured from 19–20 week-old male C57BL/N6 mice. It is assumed from the information available that all rodents were not in isolation during the duration of the experiments.

### GeneLab transcriptome analysis

The microarray experiments from all datasets mentioned were previously performed on Affymetrix platforms. An in-depth experimental detail for each dataset is available on the GeneLab website. Raw data for all studies were first background adjusted and quantile normalized using RMAExpress^11^. Data were then imported into MultiExperiment Viewer^12^ and statistically significant genes were determined by t-test with a P-value < 0.05 for all comparisons to take forward for pathway analysis. Independent and separate analysis was done for each dataset using these conditions due to platform incompatibility. Due to the small size of biological replicates available for the datasets on GeneLab we were only able to use P-value statistics. Although this might increase the chances of false positives it will also reduce the chances of false negatives. In addition, this analysis demonstrates that GeneLab datasets with less than optimal biological replicates will be able to generate a hypothesis and useful information to direct future experiments.

Next, a pathway analysis of the selected genes was performed by using a fold-change ≥ 1.2 (or ≤ −1.2) comparing AEM versus vivarium conditions. This fold-change is an arbitrary value which we believe will produce the maximum amount of the genes above the noise. One method for observing pathway relationships was done using Ingenuity Pathway Analysis (IPA) software (Ingenuity Systems). Upstream regulator analysis from IPA identified any molecule that affected the expression or function of the measured downstream target genes. The activation state of each upstream regulator from the experimental data set was determined by calculating the z-score ( ≥ 2, activated or ≤ −2, inhibited). Similar analysis through IPA was done predicting biofunctional activity. Gene set enrichment analysis (GSEA) was performed using the entire list of genes and with leading edge analysis as previously described by Subramanian *et al*^13^. Significant gene sets between age groups were considered with false discovery rate (FDR) of 0.05. Network representation of GSEA functions was done using Cytoscape.

An unbiased method to identify key genes/drivers were determined as previously reported in Beheshti *et al*^8^. by locating the overlapping genes involved in predicting significant upstream regulators, biofunctions, and GSEA gene sets (which includes the following gene sets: C2, C5, and hallmarks gene set). More specifically, for each set of genes under analysis, the association with statistically significant pathways and functions were determined through both IPA and GSEA. Common genes were determined to be involved in the analysis of both IPA upstream regulators and biofunctions. These sets of genes were further compared to the GSEA’s leading edge genes (FDR < 0.05). The overlapping genes between these two analyses were considered to be the key genes involved and in control of the majority of predicted functions and activity with the system being analyzed. In previous publications, we have validated key genes determined with this method through experimental approaches involving Western blots, qPCR, and other functional methods^8,14^. These experiments proved that the key genes determined with this bioinformatics interrogation method were indeed involved in the system being studied.

## Results

### Comparing transcriptome changes between AEM and vivarium ground controls of rodent spaceflight experiments

We selected four rodent (3 mouse, 1 rat) spaceflight studies^2,3,9,10^ that used both AEM and vivarium housing (Fig. 1B) as ground controls, and compared global transcriptomic changes associated with different housing conditions. We used publicly available GeneLab data collected from different tissues (Fig. 1) including liver^10^, muscle^2,3^, and mammary gland^9^. The liver (GLDS-25) and skeletal muscle (GLDS-21) datasets were generated from 11 week old C57BL/6 female mice, while the Bion-M1 (BF) muscles were from 19–20 week old C57BL/6 male mice. The mammary gland tissue was from female Sprague-Dawley rats.

**Figure 2:**
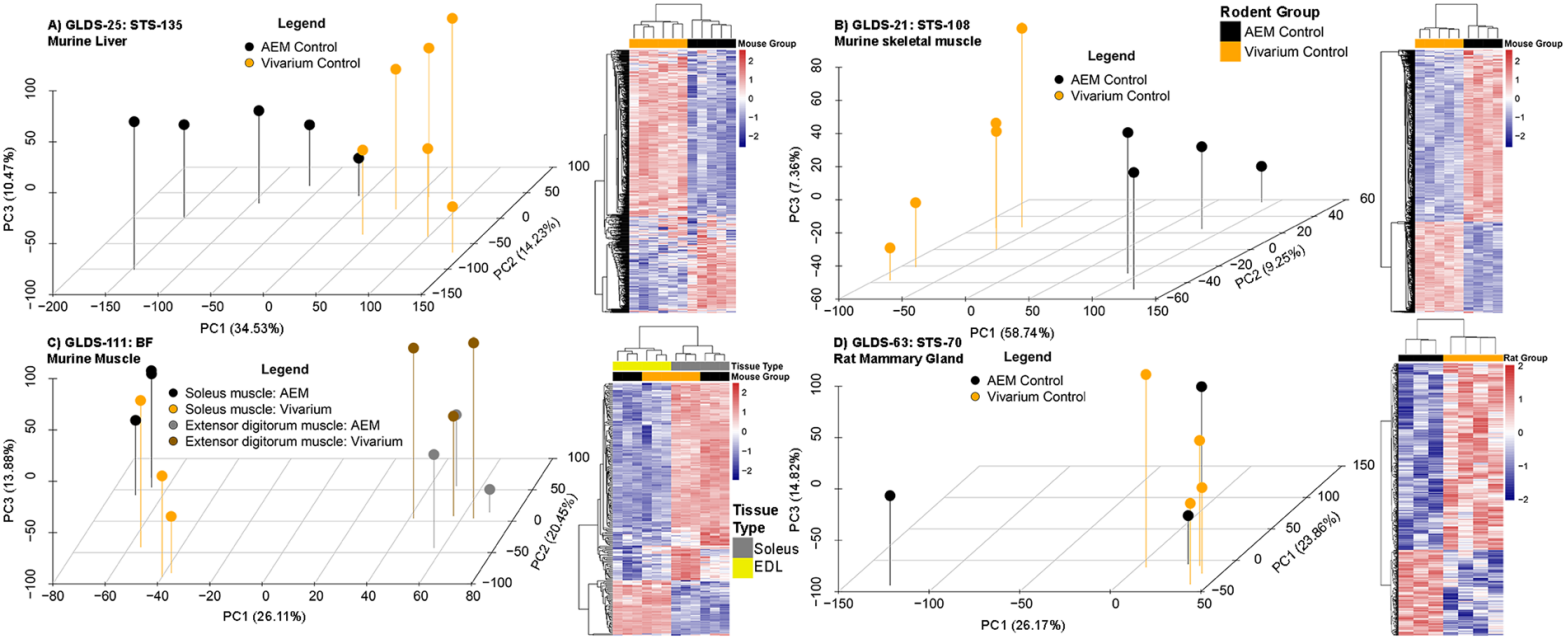
Global transcriptome analysis comparing tissue from rodents in AEM vs vivarium housing. Principal component analysis (PCA) and heatmaps representing hierarchical clustering of significantly regulated genes by complete linkage and Euclidean distance calculation (with either p-value < 0.05 or FDR < 0.05) for the following GeneLab datasets and tissue: (**A**) GLDS-25/STS-135 murine liver, (**B**) GLDS-21/STS-108 murine skeletal muscle, (**C)** GLDS-111/BF murine muscle (soleus and extensor digitorum longus), and (**D**) GLDS-63/STS-70 rat mammary gland. The percentage variance is shown for each PC in parenthesis next to PC axis.

Global differences were observed in tissue from rodents in AEM and vivarium housing (Fig. 2 and Supplemental Fig. 1). Principal component analysis (PCA) demonstrated obvious differences between individual AEM and vivarium samples for all conditions (Fig. 2A–C) except for mammary glands from rats (Fig. 2D). It is apparent from the percent variance in PC1 that clear separation occurs between the vivarium and AEM samples (especially for the GLDS-21 skeletal muscle tissue with 59% variance in PC1). Once we determined the significantly regulated genes (p-value < 0.05 or FDR < 0.05*)*, clear differences were observed between AEM and vivarium conditions for all datasets as indicated by the clear division between the up (red) and downregulated (blue) genes represented in the heat maps (Fig. 2 heat maps). Lastly, we analyzed the significantly regulated genes for each dataset with Silhouette plots through K-means statistics (Supplemental Fig. 1). This revealed distinct population of genes which have some functional impact for each dataset. Interestingly, for both Extensor Digitorum Longus (EDL) and Soleus muscles the two functions related with one cluster of genes are shown in the literature to be present with higher regulation of CO_2_ in the environment^15^. This indicates that systematic biological differences occur in the tissue of rodents depending on the housing.

### Rodent housing conditions influence the transcriptomic pathways that regulate metabolism and immune responses

**Figure 3:**
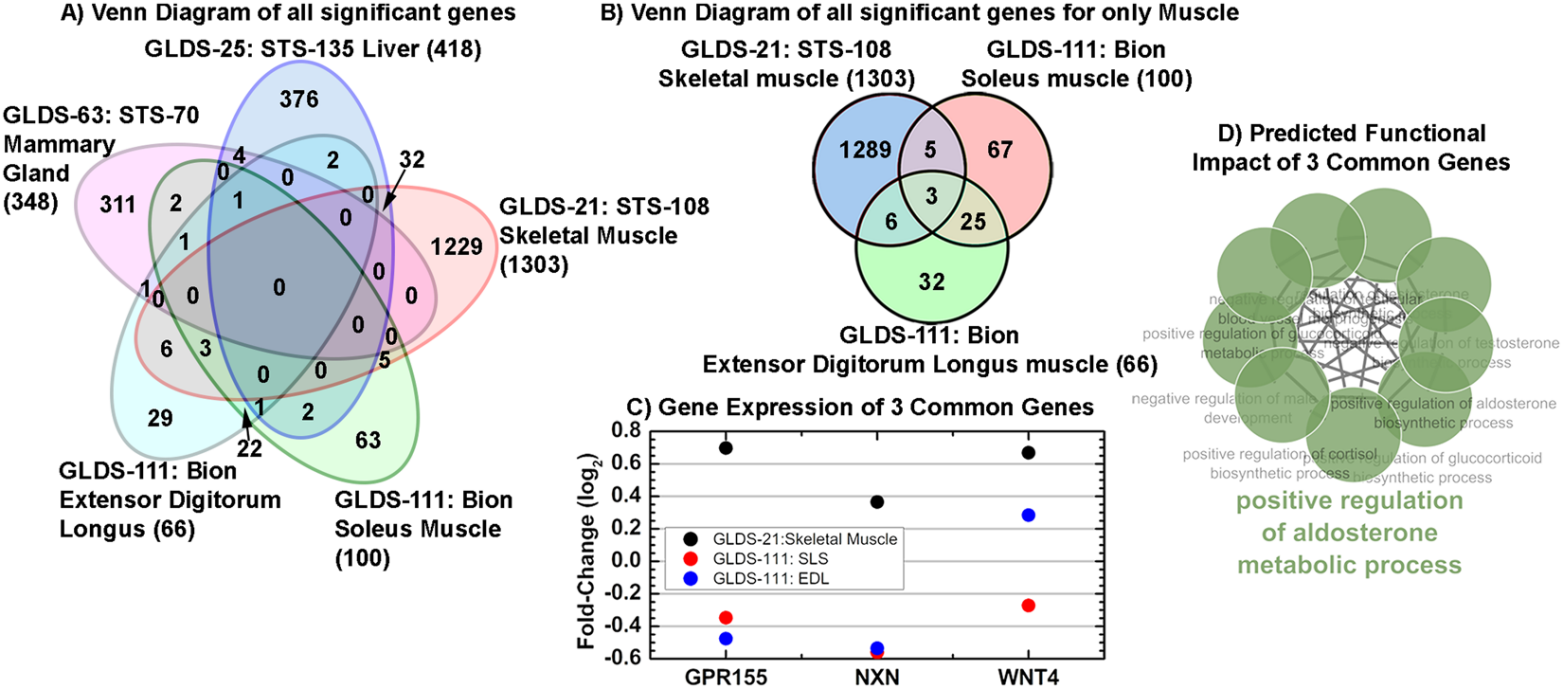
Comparison of significantly regulated genes between AEM vs vivarium housed rodents. (**A**) Venn diagram of significantly regulated genes with 1.2 fold-change for AEM vs vivarium house rodents for all tissues. (**B**) Venn diagram of significantly regulated genes with 1.2 fold-change for AEM vs vivarium house rodents for only datasets containing muscle tissue. (**C**) The fold-change values for the 3 common genes between the 3 muscle tissues. (**D**) The predicted functions of the 3 common genes determined through ClueGO^16^. The overall common function is shown with the bold green text.

By utilizing established systems biology techniques, we determined the specific biological pathways and key drivers responsible for the systematic differences observed due to housing. We analyzed each dataset separately to identify significantly regulated genes, since all results were generated by microarray assays performed on different Affymetrix platforms (the analysis methods are described further in Materials and Methods). The significantly regulated genes from each dataset were then compared to each other to find possible overlap of genes that might be in common when comparing AEM *vs*. vivarium conditions (Fig. 3). There was no overlap of significantly regulated genes between all the different tissues (Fig. 3A), which was not surprising given the differences in functions of sampled tissues. However, by focusing on muscle, which was the only tissue type used in multiple experiments, we identified three housing-dependent genes that were differentially expressed in all three types of muscle (skeletal, soleus and extensor digitorum longus) in two studies, STS-108 and Bion-M1 (Fig. 3B): *GPR155* (G-protein coupled receptor 155), *NXN* (nucleoredoxin) and *WNT4* (wingless-type MMTV integration site family, member 4). Notably, gene expression in two muscles from the same GLDS-111/Bion-M1 study followed a similar pattern that was distinct from the muscle in GLDS-21/STS-108, which suggested a study-dependent effect (Fig. 3C).

The functional impact of these three genes in association of AEM environment was predicted by ClueGO^16^ to be commonly regulated by the aldosterone metabolic process (Fig. 3D). Aldosterone is a mineralocorticoid hormone that regulates ion balance, kidney and liver function, and cardiovascular inflammation^17^. An increase in aldosterone is associated with metabolic syndrome, which is characterized by chronic inflammation, and aldosterone secretion can be triggered by hypoxia^18^. Further investigation is needed to validate aldosterone as a master regulator and to understand in which direction its metabolic process is regulated by AEM housing. However, based on our analysis we can generate a hypothesis that the association between AEM and inflammatory pathways is mediated by an increase in aldosterone due to a mild hypoxic environment in AEM compared to vivarium cages.

**Figure 4:**
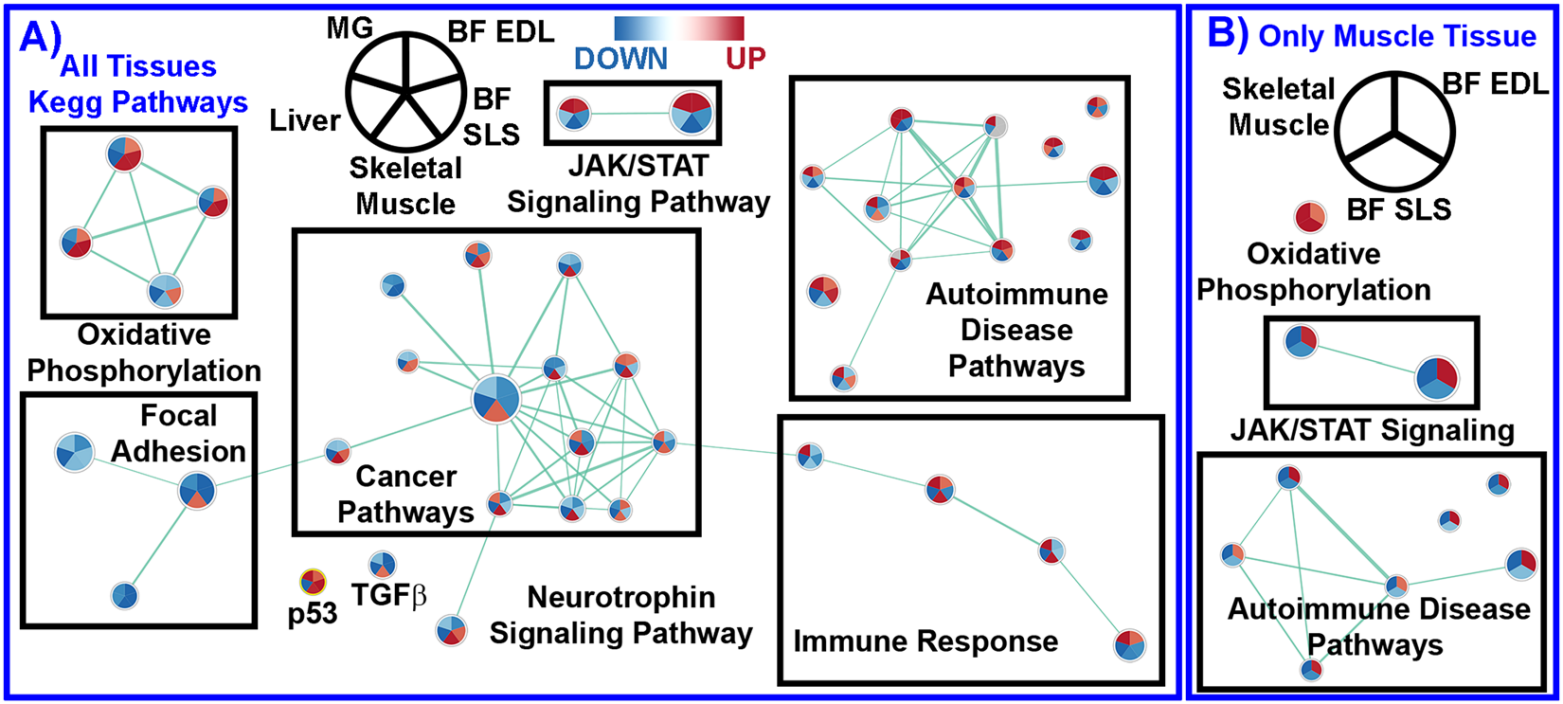
Partial network representation of gene sets listed in the KEGG pathway database from GSEA C2 gene set annotations. The GSEA network only shows the most relevant pathways. The complete network of KEGG pathways is shown in the Supplemental Fig. 2. (**A**) The statistically significant gene sets and the overlapping regulation of each gene set for all datasets and tissues. (**B**) The statistically significant gene sets and the overlapping regulation for only muscle related tissues. Leading edge analysis with an FDR < 0.05 determined significant gene sets enriched for each group. The size of each node reflects the amount of molecules involved in each gene set. The edge (green lines) represents the number of genes associated with the overlap of two gene sets (or nodes) that the edge connects. Clusters were named according to common function in each grouping. Upregulated gene sets were denoted with red color and downregulated gene sets were denoted by blue color. The grey color represents no change in regulation for that dataset. The legends on the top show which quadrant in the node is associated with a specific tissue with BF EDL = Bion, Extensor Digitorum Longus, BF SLS = Bion Soleus Muscle, and MG = Mammary Gland. The labels for the majority of single nodes without connections were not shown.

**Figure 5:**
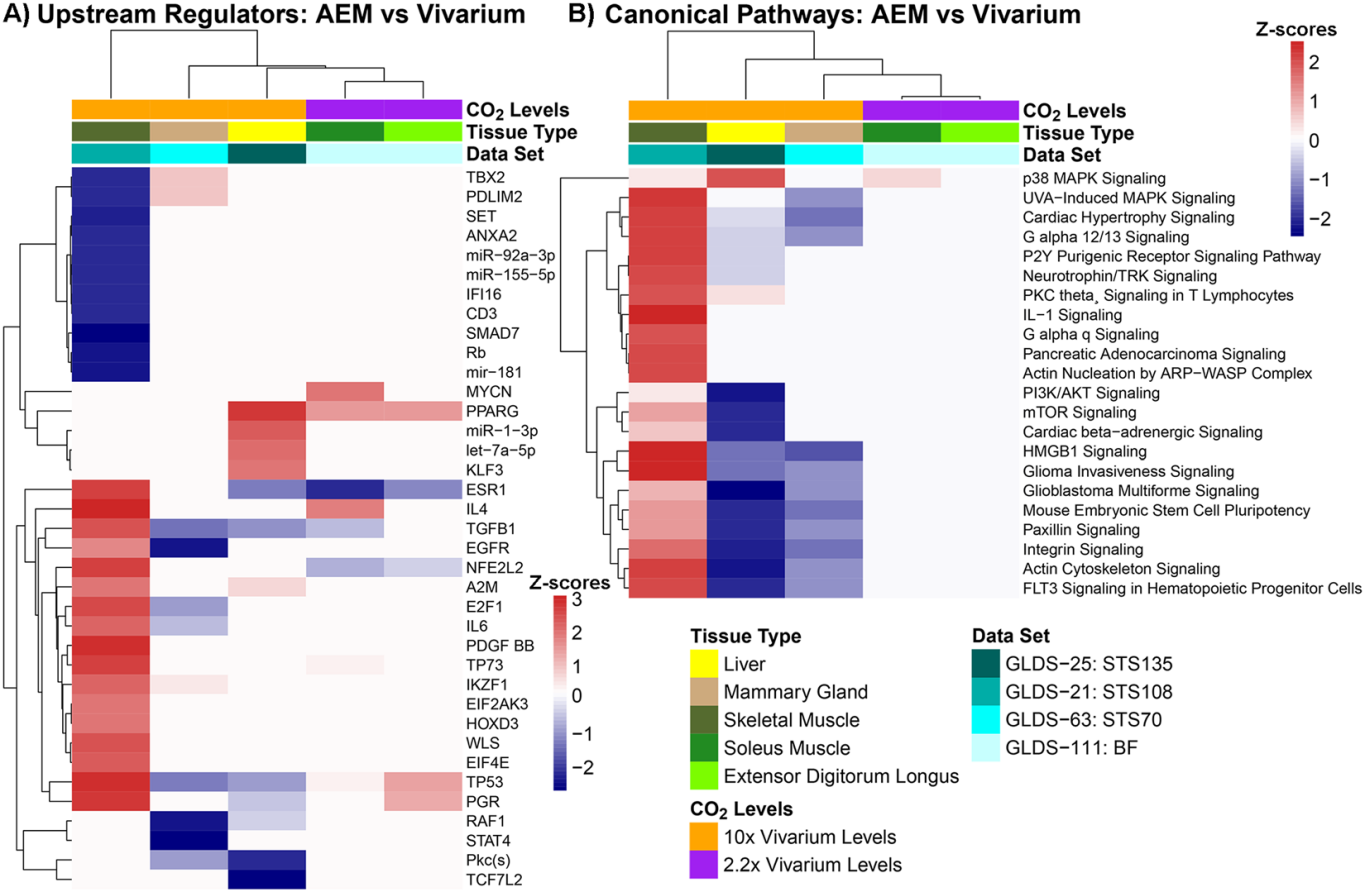
Upstream regulator and canonical pathways predictions for AEM vs vivarium housed rodents. Hierarchical clustering based on the Z-scores used to predict either activation (Z-score > 0) or inhibition (Z-score < 0) for predicted upstream regulators (**A**) or canonical pathways (**B**).

The global functional impact of AEM *vs*. vivarium cages on rodents was determined by utilizing Gene Set Enrichment Analysis (GSEA) with Kegg Pathway Gene Sets^13^ (Fig. 4), upstream regulator predictions through Ingenuity Pathway Analysis (IPA) (Fig. 5A), and canonical pathways prediction also through IPA (Fig. 5B). IPA predictions were done by utilizing Z-score statistics with Z-scores ≥ 2 indicated activation while Z-scores ≤ −2 indicated inhibition. Using multiple pathway analysis tools allowed us to find central and key pathways that are being affected when comparing AEM with vivarium housing. It was revealed that tissue specific effects occur with AEM vs vivarium housing (Fig. 4A and Supplemental Fig. 2A). For example individual datasets revealed more functional similarities in the muscle transcriptome from mice housed in AEM *vs*. vivarium cages, including an upregulation of oxidative phosphorylation combined with a downregulation of cytokine-receptor interactions, complement cascades and JAK/STAT pathways, suggesting worse cellular damage and abnormal immune responses in AEM-housed mice (Fig. 4B and Supplemental Fig. 2B). Furthermore, AEM was associated with increased immune activation in the mammary gland as well as inhibited metabolism and cell cycle components in the liver (Fig. 4A and Supplemental Fig. 2A), emphasizing that the effects are tissue-specific.

When comparing multiple pathway tools we were able to observe that p53 and TGFβ1 were altered in AEM conditions in the majority of the rodents (Figs 4A and 5A)^19,20^. Out of these factors, TGFβ is the only one strongly involved in inflammation^21^ and oxidative stress responses^22^, and is known to be regulated by hypoxia. TGFβ1 was downregulated for all tissues except for the skeletal muscle (and did not appear in the Extensor Digitorum Longus (EDL)), while p53 was up-regulated in the EDL and skeletal muscle and downregulated in liver. We also identified multiple canonical pathways that were strongly altered in high-CO_2,_ but not low CO_2-_matched AEM environment (Fig. 5B). Interestingly, most of them were upregulated in skeletal muscle (Fig. 4B, Supplemental Figs 2B and 5B), but downregulated in liver and mammary gland (Fig. 4A, Supplemental Figs 2A and 5b), emphasizing that environmental effects appear organ-specific.

### Identifying key cage-dependent transcriptional drivers

An unbiased systems biology analysis was utilized to determine the key drivers of housing-dependent changes^8,14^. As previously reported^8,14^, the key drivers were determined by finding the overlapping genes which were involved predicting the GSEA functions, IPA Upstream regulators, Canonical pathways, and Biofunctions. These common genes can be thought of as the central drivers controlling many functions in the system that is being studied. As previously shown for other experiments, once these newly determined drivers are knocked-out, most of the functions will not be activated and the system will be deficient^14^.

**Figure 6:**
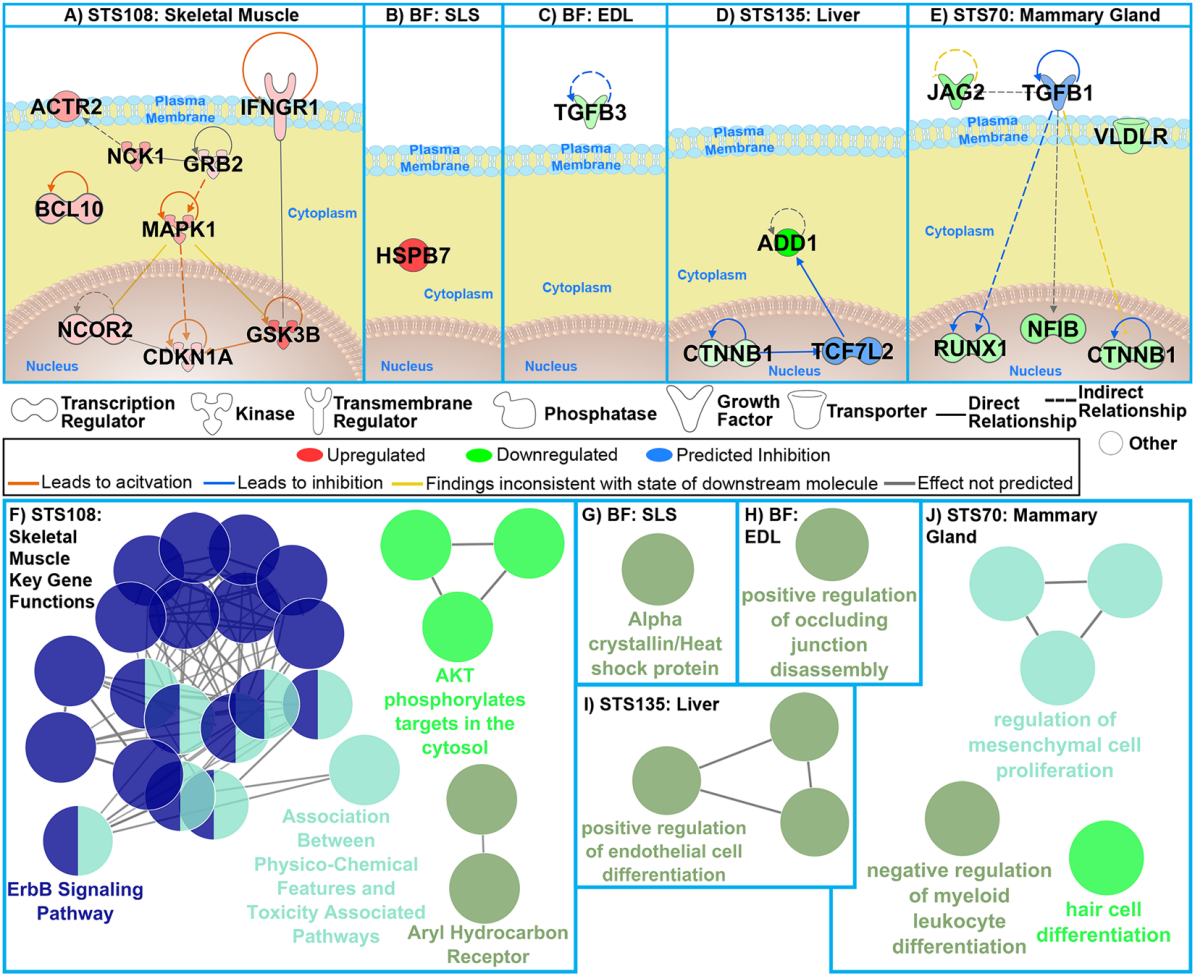
The impact of predicted key genes for tissue from rodents in AEM vs vivarium housing. (**A**–**E**) Network representation of key genes for each GeneLab dataset. The predicted relationships between all genes are also shown. Log_2_ fold-changes (with a cutoff of 1.2 fold-change) to the gene expression were used to obtain different shades of green for fold-change in downregulated genes, while different shades of red depict fold-change in upregulated genes. The darker the shade of green or red, the greater the fold-change. TCF7L2 and TGFβ1 were added to the networks to provide connectivity and the predicted regulation based on the key genes is shown to inhibit both genes. (**F**–**J**) The predicted functions for the key genes for each dataset determined through ClueGO. Each node (circle) represents a specific predicted function with the colors representing the common predicted pathway shown by the text with the same color. Nodes which are associated with more than one common predicted function is shown as two colors.

Consistent with prior results, the driver genes in the muscle and the mammary gland (Fig. 6A–C,E) were associated with inflammation (e.g. NCK1, BCL10, IFNGR1, TGFβ1, JAG2, VLDLR)^21,23–26^, and oxidative stress (NCK1, GSK3B, TGFβ1)^22,23,27^. TGFβ1 was added as the driver that provided the most connectivity between other genes in the network. Its expression was significantly different between AEM and vivarium controls across multiple tissues, and its expression is known to be associated with multiple spaceflight-relevant conditions and altered by hypoxia (Figs 4 and 5). For the liver (Fig. 6B) the two key drivers were ADD1 and CTNNB1 and a connection was made through TCF7L2. TCF7L2 was predicted to be the most statistically relevant gene that connects these two key genes. It was predicted that the influence of the two key liver drivers will inhibit TCF7L2. Interestingly decrease in TCF7L2 has been shown to have direct impact on hepatic glucose metabolism with the absence of TCF7L2 in the liver causing promotion of blood glucose levels and increased glucose intolerance^28,29^. Also, through predicted upstream regulator analysis both TGFβ1 and TCF7L2 were also predicted to be significantly inhibited (Fig. 5A) for the specific tissue.

The key liver genes indicated AEM-dependent reduction in metabolism (ADD1)^30^ and unexpectedly, reduced carcinogenesis via inhibition of CTTNB1, which is a major tumorigenic factor. On the other hand, we identified multiple pro-tumorigenic factors, such as NCK1, BCL10, IFNGR1, NCOR2, JAG2, TGFβ1, VLDLR, NFIB, RUNX1^19,31–33^ and CDKN1A/p21^34^, to be linked with AEM housing in skeletal muscle and in mammary gland (Fig. 6). Several driver genes were associated with a major carcinogenic p53 pathway (CDKN1A, HSPB7) and autophagy (BCL10), which is necessary for both immune regulation and tumor progression. Notably, a large proportion of the genes that were altered by cage type across multiple tissues (e.g. TGFβ1, GSK3B, TGFβ3, CTNNB1, CDKN1A, JAG2 and VLDLR) are regulated by hypoxia^35–39^. Furthermore, the main pathways associated with the key driver genes primarily regulate immune responses (AKT-dependent phosphorylation^40^, aryl hydrocarbon receptor^41^, alpha crystallin^42^, myeloid leukocyte differentiation^43^) and vascular morphology (occludin, endothelial cell differentiation^44^) (Fig. 6F–J), and interact with TGFβ^40–44^. The immune responses were also shown to be globally regulated from the GSEA analysis (Fig. 4 and Supplemental Fig. 2) which indicates that the overall immune response can be driven by these key genes. These results suggest that flight-matched AEM environment targets systemic immunity, via TGFβ signaling.

## Discussion

Using global transcriptome analysis of publicly available GeneLab data from multiple spaceflight studies, we were able to determine key biological differences associated with rodent environmental habitat (spaceflight-matched AEM *vs*. vivarium cages) in liver, muscle and mammary gland tissues. An established unbiased systems biology approach^8,14^ was used to determine the impact of rodent habitats on transcriptional pathways related to immune regulation, metabolic activity and tumorigenesis.

Although prior studies have analyzed behavioral and physiological responses in AEM housing, our study is the first to compare rodent adaptation to spaceflight-matched and standard ground environment at the transcriptional level^45^. A recent comparison of anatomical and physiological parameters of mice and rats housed in AEM and vivarium cages on ground (without a matching flight experiment)^45^ indicated physiological changes associated with immune and metabolic functions. For example, AEM-housed rodents had enlarged spleens and increased cholesterol. The duration of this experiment, 35 days, was significantly longer than our studies (~13 days), which may have exacerbated AEM cage effects, but it is representative of current rodent research on the ISS.

Based on IACUC examination at NASA ARC, AEM-housed rodents showed no visible or physiological signs of stress compared to vivarium-housed controls. On the other hand, there have been no studies explicitly comparing stress responses at specific tissue levels for vivarium-housed controls. Chronic low-grade stress in AEM would be consistent both with increased systemic inflammation and reduced thymus^45^. However, contrary to the “stress hypothesis”, AEM allows housing more mice per cage, and higher housing density in mice tends to be associated with lower heart rate and other signs of reduced stress^46^.

Metabolic pathways were consistently shown to be altered in the tissues between AEM vs vivarium housed rodents (Figs 3–6). Cage-dependent differences in metabolism could be caused by different nutritional content of food or different metabolic responses to the same food. Flight food primarily differs from vivarium food in its consistency: microgravity conditions require non-crumbing solid food bars, irradiated and treated with potassium sorbate to prevent mold and bacterial growth^47^. However, nutritionally flight and vivarium diets are almost identical except for vitamin content, which tends to be higher in the flight diet except for vitamin K^47^ and a few differences in indispensable amino acids, including a reduction in methionine^47^. Although vitamin K has been inversely associated with inflammatory markers in an epidemiologic study^48^, and methionine and choline deficiency combined with high fat diet is used to cause liver inflammation in rodent models, these results were achieved by the complete absence of nutrients instead of a minor reduction, so the dietary effects are unlikely to play a major physiological role in AEM vs. vivarium environments.

Furthermore, any changes in diet or exercise can alter the microbiome, which has been shown to regulate both metabolism and immune responses^49^, and since mice are coprophagic, the microbiome is directly shared between cagemates. In the limited number of investigations of microbiome responses to spaceflight environment, the microbiome has been compared between flight and ground AEM-housed rodents, but not between AEM and vivarium. Thus, it may be advisable to include microbiome as a routine component for future studies.

We believe that the most plausible explanation accounting for housing related differences in rodents relates to carbon dioxide level differences between AEM versus vivarium cages. The concentration of carbon dioxide is slightly increased in AEM ground control cages to match the flight conditions. Although the CO_2_ concentration in AEM cages on ground or in spaceflight does not exceed 0.6%, which is the maximal permitted level, its concentration in shuttle-matched AEM is approximately 10-fold higher and in Bion-M1-matched AEM is approximately 2-fold higher than the typical ground concentration. Increased CO_2_ levels could cause either chronic hypoxia, or hypercapnia and mild acidosis^28^.

Mild chronic hypoxia or hypercapnia due to increased CO_2_ levels could explain both the increase in immune responses and a reduction in metabolism, especially since the key driver genes associated with changing the housing conditions, including GSK3B, CTNNB1, JAG2, VLDLR and CDKN1A have also been reported to respond to hypoxia^35–39^. In general, a combination of reduced metabolism and systemic immune dysfunction that is predicted based on transcriptomic levels in AEM cages could contribute to abnormal responses to stressors, including infection, and play a role in systemic decline in organ functions in a manner that resembles the aging process. These deficits in metabolism and immunity can be caused by multiple factors specific to the cage environment, such as the changes in gas composition leading to a mild CO_2_-dependent hypoxia/hypercapnia^50^. Furthermore, the relatively few transcriptional changes associated with Bion-M1-matched AEM could be caused by a lower increase in CO_2_ compared to shuttle-matched AEM. However, it is important to emphasize that the causal relationship between CO_2_ levels and these physiological outcomes remains to be confirmed or rejected based on experimental evidence.

Chronic CO_2_ exposure *in vitro* (10%, while standard cell culture media is kept at 5%) has been reported to induce inflammation via NFkB pathway upregulation^51^, although it is unclear whether this result would translate to the whole organism. There are conflicting reports on rodent responses to mild increases in CO_2_ *in vivo*. Increasing CO_2_ to 3000 ppm has previously been demonstrated not to affect rat physiological parameters with the exception of sodium levels;^52^ however, increasing it to 2000–4000 ppm in mice induces systemic inflammatory responses and vascular leak in muscle^53^. Notably, neither of these studies performed a transcriptional-level analysis. Our results suggest that in order to fully examine the effect of CO_2_ it would be advisable to study the responses at the transcriptional level to different CO_2_ concentrations by keeping the rest of the environment constant (e.g. only using AEM cages).

In conclusion, through a systems biology approach we observed global transcriptomic changes in rodents induced by spaceflight-matched environment in AEM cages. We also identify spaceflight CO_2_ levels as a potential environmental stressor that merits experimental investigation. More generally, systematically changing one environmental aspect at a time (gas concentration, radiation, microgravity, etc.) and analyzing and comparing transcriptional responses could be used to create a network that could predict the most relevant causes and countermeasures for spaceflight-associated conditions, as well as confounding factors for spaceflight experiments.

Finally, our work shows how one can generate new hypothesis utilizing a portion of raw experimental data available to the public in GeneLab platform. Our results indirectly suggest that before designing future experiments it is valuable to access publicly available datasets, such as GeneLab, in order to examine potential confounding factors from spaceflight conditions. This work also highlights that metadata completeness is critical in order to better identify these confounding factors, such as carbon dioxide levels. These specific results were limited to the data that was available on GeneLab which only included a small number of subjects. Although we were able to generate statistically significant results from these small numbers, there are limitations which occur when considering subject-to-subject heterogeneities, study-to-study variations, and tissue-to-tissue variations. For future studies investigators should consider increasing the number of replicates for each experimental condition to allow for better statistics and generation of additional hypothesis for future analysis on GeneLab.

Finally, similar studies could easily be conducted to compare the effects of spaceflight and housing/growing conditions on transcriptional changes in other model organisms such as *Drosophila* and *Arabidopsis*, which also require special hardware in flights. More generally, meta-studies using GeneLab data may inform experimental and engineering goals: from cage design to targeting a particular gene that was discovered to be significant across a variety of studies. Ultimately, integrated analyses are aimed at conserving previous resources in spaceflight, including time, effort, cost of course the crew members.

## Electronic supplementary material

Supplementary Figures and Table
