# Supplementary material for: Global transcriptomic analysis suggests carbon dioxide as an environmental stressor in spaceflight: A systems biology GeneLab case study
Source: Sci Rep. 2018 Mar 8;8:4191. doi: 10.1038/s41598-018-22613-1 (PMC5843582; doi:10.1038/s41598-018-22613-1)
Supplement: Supplementary file 1 — Supplementary Figures and Table [file 41598_2018_22613_MOESM1_ESM.pdf]

Supplementary Material

Global transcriptomic analysis suggests carbon dioxide as an environmental stressor in spaceflight: A systems biology GeneLab case study

Afshin Beheshti[1], Egle Cekanaviciute[2], David J. Smith[3], Sylvain V. Costes[3]

***share equal first authorship**

[1]Wyle Labs, Space Biosciences Division, NASA Ames Research Center, Mountain View, CA

[2]Universities Space Research Association, Space Biosciences Division, NASA Ames Research Center, Mountain View, CA

[3]NASA, Space Biosciences Division, NASA Ames Research Center, Mountain View, CA

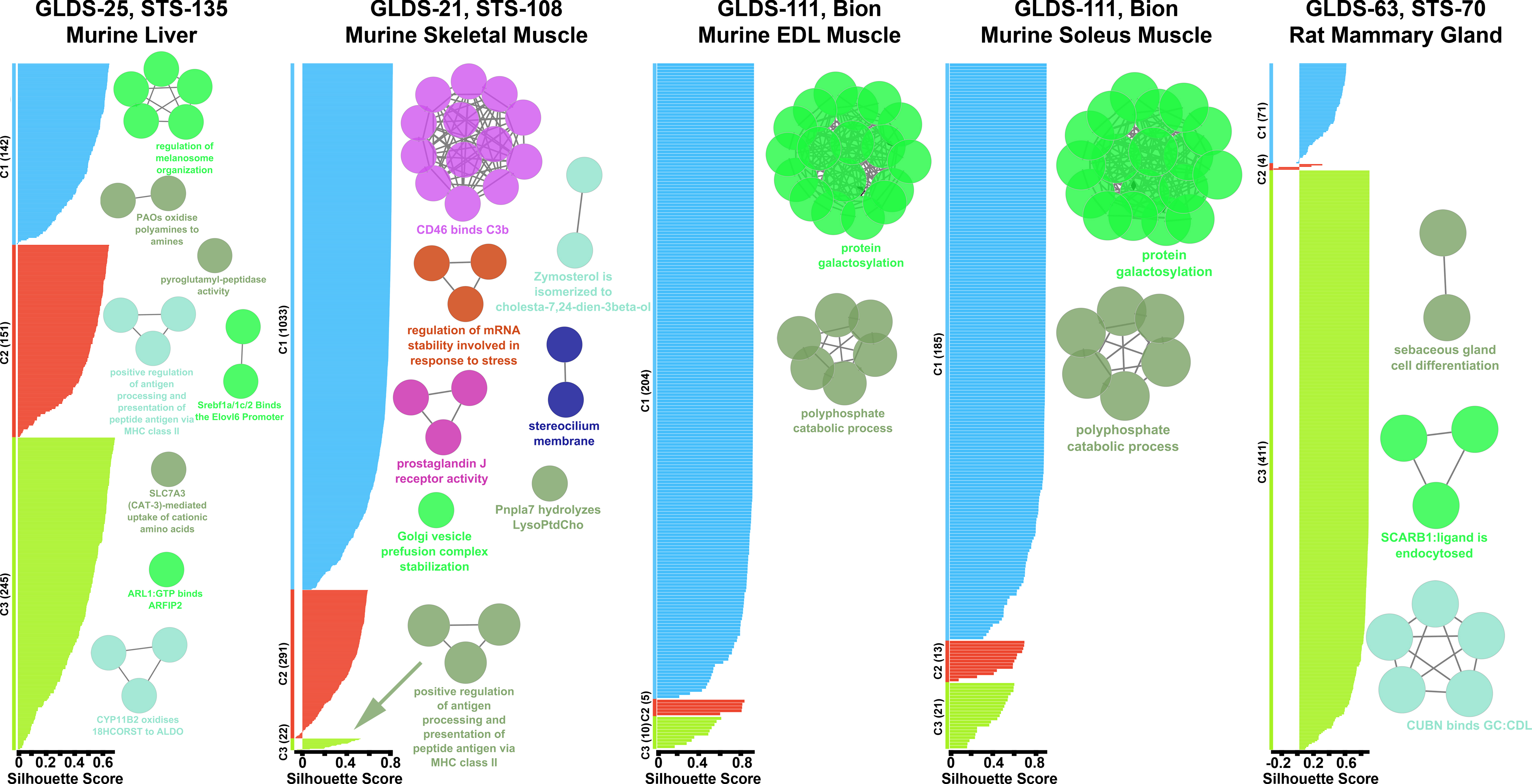
GLDS-25, STS-135
Murine Liver
C1 (142)
C2 (151)
C3 (245)
regulation of melanosome organization
PAOs oxidise polyamines to amines
pyroglutamyl-peptidase activity
positive regulation of antigen processing and presentation of peptide antigen via MHC class II
Srebf1a/1c/2 Binds the Elovl6 Promoter
SLC7A3 (CAT-3)-mediated uptake of cationic amino acids
ARL1:GTP binds ARFIP2
CYP11B2 oxidises 18HCORST to ALDO
0 0.2 0.4 0.6
Silhouette Score
GLDS-21, STS-108
Murine Skeletal Muscle
C1 (1033)
C2 (291)
C3 (22)
CD46 binds C3b
Zymosterol is isomerized to cholesta-7,24-dien-3beta-ol
regulation of mRNA stability involved in response to stress
stereocilium membrane
prostaglandin J receptor activity
Pnpla7 hydrolyzes LysoPtdCho
Golgi vesicle prefusion complex stabilization
positive regulation of antigen processing and presentation of peptide antigen via MHC class II
0 0.4 0.8
Silhouette Score
GLDS-111, Bion
Murine EDL Muscle
C1 (204)
C2 (5)
C3 (10)
protein galactosylation
polyphosphate catabolic process
0 0.4 0.8
Silhouette Score
GLDS-111, Bion
Murine Soleus Muscle
C1 (185)
C2 (13)
C3 (21)
protein galactosylation
polyphosphate catabolic process
0 0.4 0.8
Silhouette Score
GLDS-63, STS-70
Rat Mammary Gland
C1 (71)
C2 (4)
C3 (411)
sebaceous gland cell differentiation
SCARB1:ligand is endocytosed
CUBN binds GC:CDL
-0.2 0.2 0.6
Silhouette Score

Supplemental Fig 1. Silhouette plots through K-Mean Statistics for statistically regulated genes for each dataset. Generation of Silhouette plots with K-mean statistics was performed on each dataset for genes that were statistically relevant (p-value < 0.05) (genes from heatmaps in **Fig. 2**). Next to each cluster of genes the functional impact was determined using ClueGO.

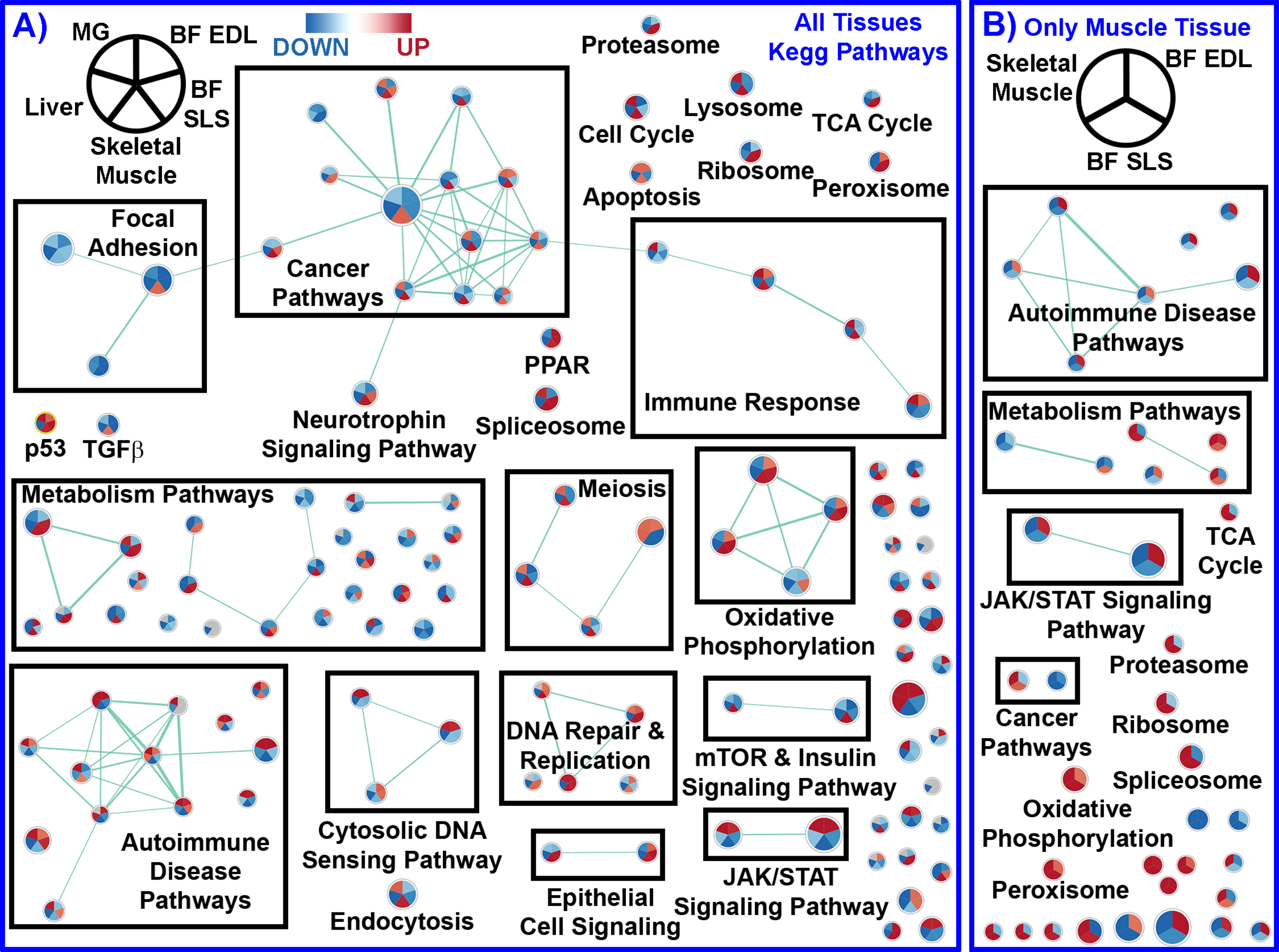

A)
MG
BF EDL
BF SLS
Skeletal Muscle
Liver
DOWN
UP
All Tissues Kegg Pathways
Proteasome
Lysosome
Cell Cycle
TCA Cycle
Ribosome
Apoptosis
Peroxisome
Focal Adhesion
Cancer Pathways
Immune Response
PPAR
Spliceosome
Neurotrophin Signaling Pathway
p53
TGFβ
Metabolism Pathways
Meiosis
Oxidative Phosphorylation
Autoimmune Disease Pathways
Cytosolic DNA Sensing Pathway
DNA Repair & Replication
mTOR & Insulin Signaling Pathway
Epithelial Cell Signaling
JAK/STAT Signaling Pathway
Endocytosis
B) Only Muscle Tissue
Skeletal Muscle
BF EDL
BF SLS
Autoimmune Disease Pathways
Metabolism Pathways
JAK/STAT Signaling Pathway
TCA Cycle
Proteasome
Cancer Pathways
Ribosome
Spliceosome
Oxidative Phosphorylation
Peroxisome

Supplemental Fig 2. Full Network representation of gene sets listed in the KEGG pathway database from GSEA C2 gene set annotations. The full GSEA network that was shown in **Fig 4**. **A)** The statistically significant gene sets and the overlapping regulation of each gene set for all datasets and tissues. **B)** The statistically significant gene sets and the overlapping regulation for only muscle related tissues. Leading edge analysis with an FDR < 0.05 determined significant gene sets enriched for each group. The size of each node reflects the amount of molecules involved in each gene set. The edge (green lines) represents the number of genes associated with the overlap of two gene sets (or nodes) that the edge connects. Clusters were named according to common function in each grouping. Upregulated gene sets were denoted with red color and downregulated gene sets were denoted by blue color. The grey color represents no change in regulation for that dataset. The legends on the top show which quadrant in the node is associated with a specific tissue with BF EDL = Bion, Extensor Digitorum Longus, BF SLS = Bion Soleus Muscle, and MG = Mammary Gland. The labels for the majority of single nodes without connections were not shown.

GO Term	Term P-Value
positive regulation of glucocorticoid metabolic process	4.73E-05
positive regulation of glucocorticoid biosynthetic process	4.73E-05
positive regulation of aldosterone metabolic process	9.46E-05
positive regulation of aldosterone biosynthetic process	9.46E-05
negative regulation of testicular blood vessel morphogenesis	4.73E-05
negative regulation of male gonad development	4.73E-05
positive regulation of cortisol biosynthetic process	4.73E-05
regulation of testosterone biosynthetic process	9.46E-05

Supplemental Table 1. Specific Gene Ontology (GO) terms for the nodes shown in Fig. 3D determined from the 3 common genes. The p-values used to determine these GO Terms are also shown.
